# Confirmed Foodborne Hepatitis A in Saudi Arabia, 2005-2015

**DOI:** 10.7759/cureus.20878

**Published:** 2022-01-02

**Authors:** Jaber Sharaheeli, Bader Alibrahim

**Affiliations:** 1 Epidemiology and Public Health, Field Epidemiology Training Program, Riyadh, SAU

**Keywords:** cross-sectional studies, saudi arabia, food-borne, food hygiene, viral hepatitis a

## Abstract

Background

Foodborne hepatitis A has major health and economic impacts. Pathogen-specific surveillance based on laboratory findings is conducted to detect and confirm cases of foodborne hepatitis A. Foodborne hepatitis A is on the priority list of diseases in the Kingdom of Saudi Arabia (KSA).

Objectives

This study aimed to describe the characteristics of confirmed foodborne hepatitis A in the KSA from 2005 to 2015.

Methods

A cross-sectional study of confirmed foodborne hepatitis A in the KSA from 2005 to 2015 was conducted, and data collection was through retrospective chart review.

Results

The number of hepatitis A cases that have been confirmed and reported to the Ministry of Health during the study period was 11148, and the Riyadh health region had more reported cases (1353 cases; 12.1%) than any other region. The highest number of cases (2631 cases; 23.6%) was recorded in 2006, and the incidence of foodborne hepatitis A was found to be highest in the month of March (1439; 12.9%). Further, the incidence of foodborne hepatitis A was highest in the five-to-14-years age group, in male individuals, and in Saudi nationals at 59% (6556 cases), 55% (6076 cases), and 88% (9775 cases), respectively.

Conclusion

The characteristics of foodborne hepatitis A vary according to time, place, and person. These variations may reflect differences in reporting systems and in preventive measures between health regions, seasons, and habits of the Saudi population.

## Introduction

Surveillance based on laboratory confirmation of pathogens is one of the main types of surveillance, and it is an ongoing process that involves collection, analysis, interpretation, and dissemination of laboratory data [1]. According to the World Health Organization (WHO), a foodborne disease (FBD) is a "disease of infections like hepatitis A or toxic nature caused by, or thought to be caused by, the consumption of food or water" [2]. The development of several cases of a similar condition due to intake of food in common is known as an FBD outbreak (FBDO). Laboratory or clinical guidelines for the confirmation of FBDO etiology differ for chemical, parasitic, bacterial, and viral agents [3]. The burden of foodborne hepatitis A in developing and developed countries is unclear. Therefore, The World Health Organization (WHO) initiated an endeavor in 2006 to estimate the worldwide burden of FBDs [4]. Regarding other hepatitis viruses, infection with the hepatitis B virus (HBV), which is prevalent in the Middle East, affects 400 million individuals globally. Further, chronic hepatitis C virus (HCV) infection affects 170 million individuals globally, and HBV and HCV infections are global epidemics. In Saudi Arabia, although the incidence of HBV and HCV infections have declined significantly, these illnesses continue to cause considerable morbidity and death, putting pressure on the healthcare system. In an epidemiological study, it was reported that the seroprevalence of hepatitis A virus (HAV) in Saudi Arabia has decreased significantly over the last two decades [5]. According to the Saudi Ministry of Health, viral hepatitis was the second-most frequent viral disease in 2007 (after chickenpox), with about 9000 new cases detected (HBV infection at 52%, HCV infection at 32%, and HAV infection at 16%) [6].

In a study conducted to determine the seroprevalence of HAV in the school students population of three regions (Madinah, Aseer, and Qaseem), Al Faleh et al. found HAV to be more prevalent than chickenpox, HBV, and HCV. Their study also stated a significant drop in overall HAV prevalence from 52% in 1989 to 25% in 1997 to 18.6% in 2008 [7]. The prevalence of HAV varies based on a population's socioeconomic status and may fluctuate within a country depending on hygienic standards [8].

As trade and travel between countries increases, so does the risk of cross-border transmission. Therefore the fight against foodborne HAV infection requires international cooperation [9]. Data on foodborne HAV infection inform public health authorities on the type and scope of the disease to facilitate early detection of disease outbreaks and to enable them plan, execute, and evaluate food safety programs [10]. Although foodborne HAV infection is notifiable, compliance is often poor, resulting in under-reporting. Poor compliance with notifying is due to the following reasons: surveillance systems for foodborne HAV infection are mostly passive as most cases of foodborne HAV infection are mild and self-limiting, therefore, patients may not seek medical evaluation; diagnosis of foodborne HAV infection requires laboratory analysis (e.g., stool culture) to identify the causative agent, and reporting of sporadic cases is generally more complete for severe disease than for mild disease, and HAV infection has a long incubation period; therefore, its diagnosis is not likely to be confirmed in a timely fashion [11]. Foodborne HAV infection is an important public health problem in the Kingdom of Saudi Arabia (KSA). Knowledge of proper food handling and FBD risk factors is poor. Hence, to raise awareness among food handlers and general public, training and health education are required [12]. The Saudi MoH developed a manual of guidelines for the surveillance and prevention of communicable diseases, including hepatitis A. The reporting times of hepatitis A from service level to regional level and from regional level to central level are within 48 hours and within one month, respectively. The Saudi MoH shares data on communicable diseases with the WHO [13].

Food safety programs must include public health surveillance as a key component. The use of public health surveillance to trace FBDs and FBDOs, including the behaviors and situations that lead to FBDs, is crucial to our understanding and control of FBDs [14]. Surveillance data can show the prevalence of foodborne hepatitis A, including the presence and scale of an outbreak, as well as hints to the outbreak's source and contributing variables [15]. The data can also be useful for hepatitis A prevention and control, for the identification of implicated factors, and for the detection of trends and outbreaks of hepatitis A. Many factors influence decisions on the type of surveillance data to collect and the method of data collection, both of which have an impact on the data's quality and value. However, the most influential factors are the contributing factors to foodborne hepatitis A and their antecedents [16]. Hepatitis A can be prevented by vaccination [17]. Educating food handlers about the importance of avoiding food cross-contamination and practising regular and proper handwashing, as well as cleaning and decontamination of surfaces and objects that come into contact with foods frequently, is a key element in the prevention and control of foodborne hepatitis A [18]. Many WHO Eastern Mediterranean Regional Office (EMRO) countries do not have established surveillance or reporting mechanisms that are adequate for the detection and tracking of foodborne hepatitis A. Under-reporting of foodborne hepatitis is a major problem in EMRO countries, and Saudi Arabia is in a unique situation due to the pilgrimage season and Umrah. Throughout the year, large numbers of hajjis visit Makkah for Hajj and Umrah, and they get their foods from local restaurants and cafeterias [19]. However, It's difficult to tell if an observed trend is the consequence of an actual hepatitis A outbreak or just a reflection of improvements in the reporting system because reported cases of hepatitis A are dependent on disease incidence and the effectiveness of the reporting system. Seasonal fluctuation in the frequency of food poisoning outbreaks has been recorded over time, with outbreaks peaking in the hot summer months of June, July, and August [12].

This period corresponds with the summer school holidays, a time when families spend a significant amount of time away from home and rely on restaurants, canteens, and other fast-food outlets for their meals [20]. In Saudi Arabia, the burden of foodborne hepatitis A is currently unknown, and hepatitis A cases must be reported to the Communicable Disease Directorate of the MoH. The laboratory confirmation methods for hepatitis A are based on the presence of serum immunoglobulin M [13]. Viral antigens can be detected using enzyme immunoassays (e.g., direct fluorescent antibody assay, indirect immunofluorescence assay, and enzyme-linked immunosorbent assay). Nucleic acid hybridization assay is a highly sensitive and specific method of virus detection. The specimen is plotted on a nitrocellulose membrane, and the viral nucleic acid in the sample is bound with the specific antibody. A polymerase chain reaction (PCR) test is performed for the detection of HAV, and it requires reverse transcription. PCR is highly susceptible to interference by substances in environmental samples [21]. It was reported in studies that foodborne hepatitis A affects male individuals more than female individuals, is more prevalent in people over 15 years of age, and has a higher incidence during rainy seasons than during the summer [22,23].

The aim of this study was to describe the characteristics of confirmed cases of foodborne hepatitis A in Saudi Arabia from 2005 to 2015.

## Materials and methods

Study design

This is a cross-sectional study.

Study population

This consisted of all reported laboratory-confirmed cases of foodborne hepatitis A in Saudi Arabia from January 01, 2005 to December 31, 2015.

Data collection

All available data on confirmed cases of foodborne hepatitis A in Saudi Arabia from 2005 to 2015 were retrospectively reviewed. Confirmed cases of foodborne hepatitis A were reported to the Communicable Disease Directorate of the MoH.

Data analysis

For data entry and analysis, Microsoft Excel software was used, and the data was analyzed in accordance with the study objectives. Frequencies of different descriptive variables were estimated to obtain percentages. The frequencies and percentages of confirmed cases of foodborne hepatitis A and their distribution by year, month, age group, gender, health region, and nationality were calculated.

Ethical concerns

The Institutional Review Board of the Ministry of Health gave the ethical approval. Official approval was provided by the Director-General of the Communicable Disease Directorate.

The privacy of personal information is safeguarded. The authors gathered and recorded data. There were no additional people involved in the data collecting or who had illegal access to the information. The study individuals could not be identified because the data record did not contain names or any other personally-identifying information.

## Results

During the 11-year study period from 2005 to 2015, 11148 confirmed cases of hepatitis A from all 20 health regions of Saudi Arabia were reported to the Communicable Disease Directorate of the MoH.

**Figure 1:**
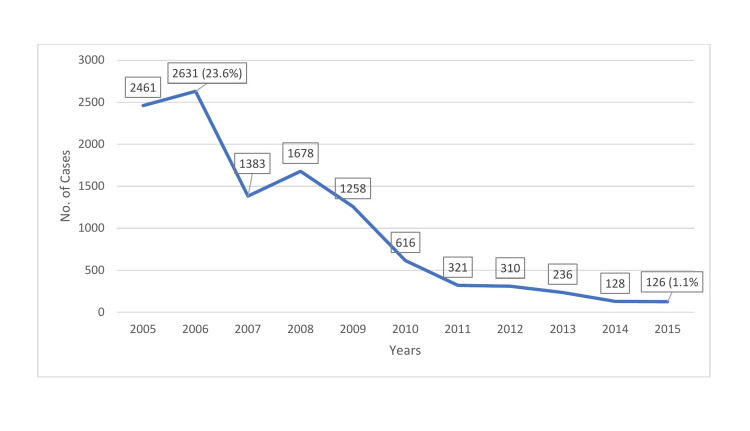
Incidence of hepatitis A by year in Saudi Arabia, 2005 to 2015

Figure 1 shows a line graph depicting the time trend of foodborne hepatitis A. In 2005, there were 2461 recorded cases of foodborne hepatitis A. The highest yearly number of hepatitis A cases was 2631 (23.6%) reported in 2006. After 2006, this number declined gradually, reaching its lowest of 126 cases (1.1%) in 2015.

**Figure 2:**
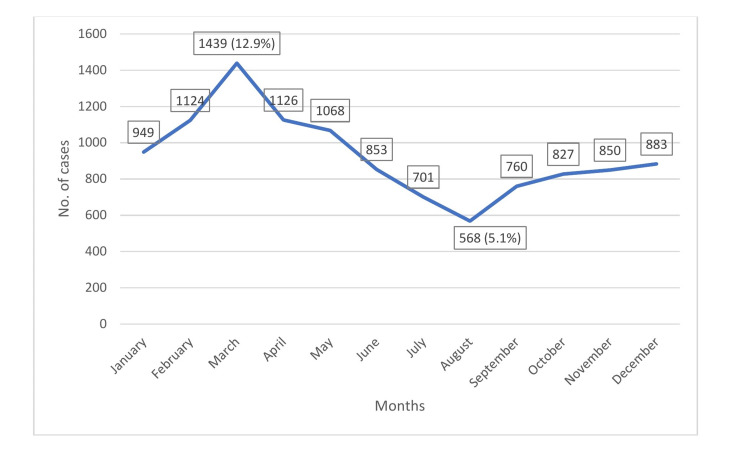
Incidence of hepatitis A by month in Saudi Arabia, 2005 to 2015

Figure 2 shows the number of hepatitis A cases by month. The number of cases increased from January until March. Hepatitis A most commonly occurred in the month of March, with 1439 of 11148 cases (12.9%) reported. After the month of March, the number of cases decreased gradually, reaching its lowest monthly level of 568 cases (5.1%) in August. Thereafter, a slight increase in the number of cases was observed.

**Figure 3:**
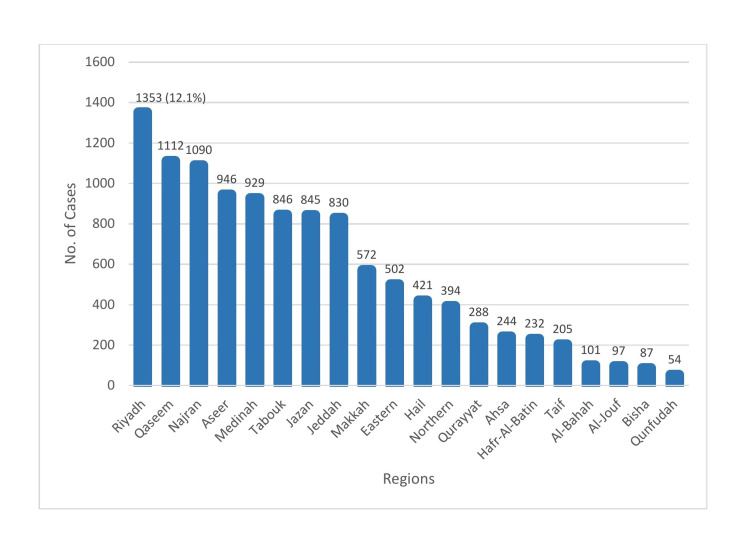
Incidence of hepatitis A by health region in Saudi Arabia, 2005 to 2015

The incidence of hepatitis A was highest in the Riyadh health region, with 1353 cases (12.1%) reported, and lowest in the Qunfudah health region, with 54 cases (0.48%) reported (Figure 3).

The incidence of hepatitis A was highest in the five-to-14-years age group, with 6556 cases (58.8%) reported, and lowest in the less-than-one-year age group, with 96 cases (0.9%) reported.

Further, at 55% (6076 cases), the incidence of hepatitis A was higher in males than in females for the entire study period, but decreased with age in both sexes.

Finally, at 88% (9775 cases), Saudi nationals had a higher incidence of foodborne hepatitis A than individuals of other nationalities.

## Discussion

During the study period of 2005 to 2015, the incidence of hepatitis A peaked in 2006. Thereafter, it declined rapidly and steadily, with slight elevation in 2008, before reaching its lowest level in 2015. This trend may be due to positive or negative factors. Positive factors include improved preventive and control measures such as good sanitation (e.g., clean water supply), better living conditions, increased awareness, more safe practices, and enhanced hepatitis A vaccination coverage. Negative factors include deterioration of detection and reporting systems (e.g., a lack of diagnostic facilities or skilled personnel). The incidence of hepatitis A was highest in March (i.e., higher during the rainy season than during the summer), and this finding is consistent with those of other studies. In a previous study, analysis of hospital-based data revealed that cholera, typhoid fever, viral hepatitis A, and dysentery (shigellosis) are the most commonly reported waterborne diseases and FBDs, with high levels of seasonality and peaks in the months of February, March, and April [23]. Outbreaks can be predicted with the onset of rains and may be a result of contamination of unprotected water sources. They may occur when people drink from contaminated water sources or when water from these sources comes in contact with food or food-contact surfaces [24,25]. In an earlier study, it was reported that season or climate does not alter disease prevalence [26].

In this study, the incidence of hepatitis A was highest in the Riyadh health region in the central province and lowest in the Qunfudah health region in the West. This finding may be due to the same reason as for the causative agents of other FBDs, which is an imbalance between preventive and control measures and the reporting capacities. In another study, marked regional variation in HAV prevalence was reported, with the highest prevalence (67%) in the northwestern region and the lowest prevalence (38.4%) in the eastern region. In most regions, rural inhabitants were affected more than urban residents [27]. The five-to-14-years age group was significantly affected by hepatitis A. This finding may be explained by delayed viral exposure, which results in a large number of susceptible adolescents and adults with low natural or acquired immunity (as a result of an absence of previous infection or low vaccination coverage, respectively), and by an increased risk of exposure to contaminated water or food [28]. The finding of high hepatitis A incidence in the five-to-14-years age group in this study differs from the finding of high hepatitis A incidence in the 15-34-years age group in an earlier study [22]. In another study, it was reported that the incidence of HAV infection is significantly high in school-age children and gradually increases with age [29]. In this study, the incidence of hepatitis A was higher in males than in females for every year of the study period, but it declined gradually for both sexes, reaching its lowest in 2014/2015. This finding is consistent with those of other studies, although one study reported no differences in disease prevalence between the sexes [22,26,27]. The incidence of hepatitis A was significantly higher in Saudi nationals than in individuals of other nationalities. This finding may be explained by the fact that Saudi nationals have a higher risk of hepatitis A exposure and are more likely to have access to healthcare than individuals of other nationalities. The Healthcare Electronic Surveillance Network (HESN) is a web-based electronic health system that was implemented in the KSA in 2012, though it was launched in 2011. HESN is a system for disease surveillance configured by the MoH of the KSA. It contains data on the management of communicable disease cases, outbreaks, and vaccination [29]. There were no differences in confirmed foodborne hepatitis A detection and reporting between the pre- and post-HESN eras. The research question was answered, and the study objective was accomplished. Although there were some missing data, the general characteristics of confirmed foodborne hepatitis A in the KSA were determined.

Limitations

Some data were missing. The distributions of cases by age group, gender, and nationality in the regions were not available. Further, there were no data on the strains of bacteria, exposure time, food items and water, exposure source, and mortality. The intervals of the age groups were not equal (e.g., every five or 10 years). For instance, the 15-44-years age group has a long interval of 29 years; hence, this age group included a disproportionately high number of cases, which adversely affected the representativeness of the age groups.

Recommendations

We recommend assessment of the reporting and surveillance systems for confirmed cases of foodborne hepatitis A and the laboratory capacities for pathogen detection, continuous training and supervision of medical staff, assessment of preventive and control measures against foodborne hepatitis A, inclusion of more information in the database for foodborne hepatitis A, upgrading the existing electronic systems to improve the quality (accuracy and timeline) of data, conducting studies to better understand the recent changes in the dietary habits of the Saudi community, and conducting further studies to better understand food safety, foodborne hepatitis A, and FBDOs in Saudi Arabia.

## Conclusions

Foodborne hepatitis A should be confirmed by laboratory analysis, and the Communicable Disease Directorate should be notified of positive cases in the health regions. Over an 11-year study period, hepatitis A was shown to be a common FBD. Significant variations in the distribution of hepatitis A were observed in the health regions and over the years under review. These variations may be due to differences in the detection, registration, and reporting systems and in preventive and control measures in the health regions. In some health regions, there may be significant flaws in the systems and measures that may warrant consideration.

Foodborne hepatitis A was found to be predominant in young male Saudi adults, which may be reflective of Saudi culture and a change in the dietary habits of Saudi nationals. The characteristics of foodborne hepatitis A in the KSA are similar to those in other geographical areas, but with some differences. The missing data would have added to our understanding of foodborne hepatitis A and the associated factors.
